# Chronic Corticosterone Administration Induced Affective Disturbances, But Did Not Alter Consolidated Active Avoidance Coping or Neuronal Activation Level, in Male Long–Evans Rats

**DOI:** 10.1523/ENEURO.0085-26.2026

**Published:** 2026-08-25

**Authors:** Zhi Hui Kang, Chun-hui Chang

**Affiliations:** Institute of Systems Neuroscience, National Tsing Hua University, Hsinchu 30013, Taiwan

**Keywords:** anhedonia, avoidance behavior, chronic stress, corticosterone, despair, hypolocomotion

## Abstract

Stress is a major risk factor for various psychiatric disorders. Activation of the hypothalamic–pituitary–adrenal axis increases cortisol release, whereas prolonged glucocorticoid exposure has been linked to cognitive impairment and mood disorders. In this study, we examined whether chronic corticosterone administration alters emotional states, active coping strategies, and associated neuronal activation in rats. Male Long–Evans rats were trained in a lever-press task to avoid signaled footshocks and then assigned into control or corticosterone-treated groups, with the latter receiving daily corticosterone injections for 21 consecutive days. Afterward, emotional states were assessed using the elevated plus maze (EPM), forced swim test (FST), and sucrose preference test (SPT). Finally, the animals were retested for their active avoidance behavior, while neuronal activation levels in related brain areas were evaluated. Our results showed that chronic corticosterone administration led to decrease in body weight and deteriorated fur condition over time. The stressed rats showed hypolocomotion in EPM but no changes in anxiety-like behavior, increased despair-like behavior in FST, and developed anhedonia in SPT. Despite these emotional alterations, active avoidance performance and freezing behavior were not affected. Consistently, analyses of numbers of c-Fos–positive neurons in the prelimbic cortex, infralimbic cortex, nucleus accumbens core, nucleus accumbens shell, dorsal CA3, and basolateral amygdala did not differ between groups. Together, our results suggested that chronic corticosterone administration induced physical deterioration. These animals were less active and developed behavioral despair and anhedonia, but did not alter their coping strategies to threat-related signals.

## Significance Statement

Chronic stress is strongly associated with affective disorders, yet previous studies have reported mixed findings on how it affects avoidance and coping behaviors. Using a chronic corticosterone administration model in rats, we demonstrated that sustained corticosterone elevation induced physiological deterioration, hypolocomotion, despair-like behavior, and anhedonia. However, well-established active avoidance behavior, along with neuronal activation levels in key corticolimbic regions, was not affected. These findings revealed a dissociation between stress-induced affective disturbances and preserved instrumental coping execution. Our results suggested that chronic corticosterone exposure may preferentially disrupt emotional processing while sparing consolidated avoidance networks, refining current understanding of how chronic stress differentially affects affective and action control systems.

## Introduction

Stressful life style and dysfunctional hypothalamic–pituitary–adrenal (HPA) axis are considered contributing environmental factors leading to various mental health conditions, including depression, anxiety, and schizophrenia (Richelson, 2001; Varese et al., 2012). While acute HPA activation facilitates adaptive responses to stress, excessive or sustained cortisol release has been associated with cognitive impairment and emotional dysregulation, suggesting that chronic HPA hyperactivation may exert detrimental effects (Charles et al., 2020). Importantly, human studies of acute and chronic stress have demonstrated substantial individual variability in HPA reactivity, in that genetic, developmental, and environmental factors influence whether stress exposure leads to adaptive coping or pathological behavioral outcomes (Kudielka and Wüst, 2010; Wang et al., 2025). These findings highlighted chronic HPA dysregulation, rather than acute activation, as a critical mechanism underlying stress-related disorders.

In rodents, corticosterone is the primary glucocorticoid and serves as the functional homolog of human cortisol (Gotlib et al., 2008; McEwen and Akil, 2020). Chronic corticosterone administration is a widely used pharmacological model to mimic stressed condition, which induces behavioral changes including heightened anxiety and depressive-like behaviors (David et al., 2009; Dieterich et al., 2019). It has been shown to worsen behaviors associated with negative valence (Gourley and Taylor, 2009; Olausson et al., 2013) as assessed through behavioral paradigms, such as the elevated plus maze (EPM) for anxiety, forced swim test (FST) for despair, and sucrose preference test (SPT) for anhedonia (Tran and Gellner, 2023). However, active avoidance, which requires a learned response to avoid punishment, is a less explored but critical aspect of adaptive fear regulation (LeDoux and Gorman, 2001; Oleson et al., 2012).

Chronic stress and prolonged glucocorticoid exposure induce remodeling within corticolimbic circuits that regulate emotional and behavioral responses (Mariotti, 2015; Kim et al., 2019). The medial prefrontal cortex (mPFC), including its prelimbic (PL) and infralimbic (IL) subregions, plays a central role in fear regulation and behavioral flexibility (Giustino and Maren, 2015; Wood et al., 2019). Repeated restraint stress induces dendritic atrophy, reduces synaptic plasticity, and disrupts the excitatory–inhibitory balance in the mPFC, whereas chronic corticosterone administration produces similar structural reorganization (Cook and Wellman, 2004; Radley et al., 2004; Yuen et al., 2012). At the subcortical level, elevated corticosterone leads to hyperexcitability of the basolateral amygdala (BLA) along with increased spine density and dendritic arborization, resulting in increased anxiety-like behaviors (Rao et al., 2012; Daviu et al., 2019). The nucleus accumbens (NAc) exhibits differential activation in response to avoidance behaviors, with hyperactivity observed during active avoidance and increased deactivation during passive coping (Levita et al., 2012). Moreover, glucocorticoid signaling has been reported to suppress active avoidance behavior and alter synaptic plasticity within the NAc, potentially affecting coping mechanisms (Gamaro et al., 1999; Garcia-Keller et al., 2021). The hippocampus is also highly sensitive to stress and glucocorticoid exposure (Zahedi et al., 2025). Chronic stress alters hippocampal plasticity, and prolonged glucocorticoid elevation induces dendritic atrophy in CA3 pyramidal neurons (Woolley et al., 1990; Pinto et al., 2015).

Active avoidance and passive coping behaviors engage distinct neural circuits. Active avoidance is primarily regulated by the NAc and mPFC, which are involved in motivation and adaptive strategies, whereas passive coping relies more heavily on the amygdala and hippocampus, which mediate fear learning and memory consolidation (Peters et al., 2009; Levita et al., 2012). Dysregulation of these circuits may contribute to maladaptive fear responses and increased vulnerability to anxiety and mood disorders. Although previous studies have examined stress effects on avoidance behavior, findings regarding shifts between active avoidance and passive coping remain inconsistent. Clarifying how chronic HPA dysregulation influences the switch between these modes is essential for understanding stress-related psychopathology.

In this study, we investigated the effects of chronic corticosterone administration on negative emotional states and selection of coping strategies under threat. The rats were trained to perform a lever-press task to avoid signaled footshocks. After reaching baseline performance, they were assigned to the control (CON) group or corticosterone (CORT) group, with the latter receiving daily corticosterone administration for 21 consecutive days. Anxiety, despair, and anhedonia were assessed using the EPM, FST, and SPT, respectively. After these behavioral assessments, the rats were retested in active avoidance tasks, and neuronal activation in the mPFC, NAc, BLA, and dorsal CA3 (dCA3) were evaluated using c-Fos immunohistochemistry. c-Fos is an immediate early gene widely used to assess neuronal activity patterns in response to behavioral tasks (Morgan and Curran, 1991). We hypothesized that chronic corticosterone administration would impair active avoidance performance while enhancing passive coping of freezing behavior, with corresponding alterations in neuronal activities in stress-related brain regions.

## Materials and Methods

### Subjects

A total of 24 male Long–Evans (LE) rats (initial weight 200–224 g; National Laboratory Animal Center, NARLabs) were used. These animals were housed at the animal facility of National Yang Ming Chiao Tung University and were kept under a 12 h light/dark cycle (8:00 A.M. to 8:00 P.M.). There was a 1 week acclimatization period for the animals before preceding to the commencement of the experiment. The experimental protocols had received the requisite approvals from the Institutional Animal Care and Use Committees of both National Tsing Hua University and National Yang Ming Chiao Tung University.

### Experimental design

In this experiment, the rats received corticosterone administration for 21 consecutive days after they successfully acquired active avoidance behavior. Afterward, the rats were tested with EPM, FST, and SPT, followed by active avoidance retest and tissue processing. The experimental design is summarized in Figure 1.

**Figure 1.**
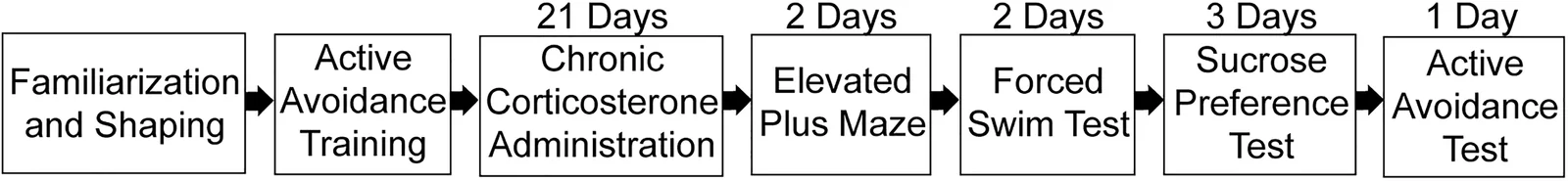
Experimental design of this study. All rats underwent a familiarization and shaping phase, followed by active avoidance training. Half of the animals then received 21 d of chronic corticosterone administration, while the other half served as controls. Afterward, the rats were tested in EPM (2 d), FST (2 d), and SPT (3 d) and finally were retested in active avoidance task on the last day.

#### Behavioral apparatus

The experiment was conducted in a behavioral room equipped with six operant chambers (Med-Associates), each fitted with a dedicated camera for recording and analyzing rat behavior. All data were collected on a computer controlling the chambers. Each chamber contained two retractable levers on the right-side wall, with cue lights positioned above them. A house light was mounted on the opposite wall. The floor consisted of 19 parallel stainless-steel rods connected to a shock generator for delivering footshocks. Beneath the grids, pans were filled with 1% acetic acid. These chambers were used exclusively during familiarization, shaping, and active avoidance sessions.

#### Familiarization and shaping

During the initial days of the experiment, all rats were introduced to the operant chambers set up with an illuminated house light but no levers or auditory stimuli presented, where the animals spent an hour for familiarization for 1 d. All rats then proceeded with a shaping procedure that lasted a maximum of 3 d. The setup included an illuminated house light and an extended left lever, where the animals spent an hour for each daily session. During shaping, each time a rat pressed the lever, it caused the lever to retract for a second before being re-extended. Progression to the next phase was contingent on a rat achieving a minimum of 40 lever presses in a single day or completing the 3 d shaping period.

#### Active avoidance procedure

The training procedure was adapted from the methods described earlier (Piantadosi et al., 2018; Capuzzo and Floresco, 2020; Kuan and Chang, 2024). Daily sessions consisted of 20 active avoidance trials with a variable 105 s (±30 s) intertrial interval (ITI). At the start of each trial, the left lever extended simultaneously with the onset of white noise stimulus (80 dB) lasting 15 s. If the rat pressed the lever within this 15 s window, it would successfully terminate the noise and retract the lever, marking the trial as “successful active avoidance.” Moreover, a successful response triggered a safety signal, which extinguished the house light and activated a stimulus light above the lever for 30 s. In cases where the rat did not press the lever within the initial 15 s, the trial moved on into the escape phase. During this phase, the sound ceased, and the rat received the first of up to three 0.5 s footshocks delivered in every 5 s. Lever pressing during this escape period would result in lever retraction, halting any subsequent shocks, activating the safety signal, and would be classified as an “escape.” If the rat failed to press the lever during the 10 s escape period, the trial would be concluded after the third shock, with the lever retracted, no safety signal being issued and house light switched off. This would be recorded as an “active avoidance failure.”

The experiment began with the shock current set at 0.2 mA. If the rat failed to meet the required performance criteria of <30% failures (out of daily 20 trials) for 3 d in a row, the shock intensity would be increased by 0.05 mA. This increase was nonreversible. Upon reaching 0.3 mA, increments were adjusted to 0.1 mA, with a maximum limit set at 0.8 mA. The performance of the rats was reassessed every time the shock intensity increased.

The rats continued this daily training until they reach <30% failures across 3 consecutive days, qualifying them as successfully acquired the active avoidance behavior. If the rats failed to learn active avoidance at a shock level of 0.8 mA, they were excluded from the study.

#### Chronic corticosterone administration

The rats were assigned into matched CON group and the CORT group after they successfully acquired lever-press active avoidance behavior. CORT rats received subcutaneous injection (40 mg/kg) of corticosterone in sesame oil containing 10% ethanol (40 mg/ml) for 21 consecutive days. CON rats were handled daily under identical conditions but did not receive injections. Injections were administered between 4:00 P.M. and 5:00 P.M. each day to minimize circadian variation. The injection volume was 1 ml/kg. The corticosterone solution was prepared weekly.

#### Fur coat state

Coat state is an indicator of grooming frequency and serves as a measure of motivation for self-care. The assessment of coat state follows the criteria described by Nollet et al. (2013), evaluating seven regions: head, neck, back, abdomen, tail, forepaws, and hindpaws. Each area is scored based on the condition of the fur: 0 (good) for smooth and shiny fur with no signs of tousling or spiky patches; 0.5 (moderate) for slightly fluffy fur with some spiky patches; and 1 (bad) for unkempt fluffy fur with slight staining.

#### EPM

The EPM was used to assess anxiety-like behavior. The apparatus consisted of two open arms and two closed arms arranged in a plus configuration and elevated 55 cm above the floor. To minimize novelty-induced stress, animals were habituated to the behavioral testing room for 2 h in their home cages on the day prior to testing. On the test day, the rat was gently placed on the central platform facing a closed arm, with video recording for 5 min. Anxiety-like behavior was quantified as the percentage of time spent in the open arms relative to the total time spent in both open and closed arms, as well as the number of entries into open versus closed arms.

#### FST

The FST was conducted to assess despair-like behavior. The apparatus consisted of a transparent cylinder (50 cm in height × 20 cm in diameter) filled with water maintained at 23 ± 1°C to a sufficient depth to prevent animals from touching the bottom. Following the EPM test, the rats underwent a 15 min pre-exposure session to facilitate adaptation to the testing procedure. On the next day, animals were placed in the cylinder for a 5 min test session, during which behavior was video-recorded. The duration of immobility was measured and used as an index of despair-like behavior.

#### Sucrose preference

The SPT was conducted to assess anhedonia-like behavior. Each rat was housed individually and provided with two bottles for 3 consecutive days. One bottle contained 250 ml of plain drinking water, and the other contained 250 ml of a 1% sucrose solution. Fluid consumption was measured over 24 h periods from 5:00 P.M. to 5:00 P.M. the following day. The positions of the bottles were alternated daily to prevent side preference. Sucrose preference was calculated as the percentage of sucrose solution intake relative to the total fluid intake.

### Histology

To ensure optimal detection of c-Fos expression, the rats were killed 90 min after active avoidance behavioral retest. They were deeply anesthetized using CO_2_ and then undergo transcardial perfusion with 100 ml of saline, followed by 150 ml of 4% paraformaldehyde in 0.1 M phosphate-buffered saline (PBS). Their brains were extracted and postfixed for not longer than 1 d and then cryopreserved in 25% sucrose in 0.1 M PBS. These sections were subsequently used for the immunohistochemical analysis of nuclear c-Fos protein. The brains were sectioned coronally by 35 μm, collected sequentially into six adjacent sets, and stored in cryoprotectant at −20°C. One tissue section series (containing sections spaced by 210 μm) from each rat was mounted onto gelatin-coated slides for anatomical reference (Nissl staining), and another section series was used for later immunohistochemical processing.

### Immunohistochemistry

The c-Fos immunoreactivity was detected using a standard avidin–biotin peroxidase method. Free-floating sections were incubated overnight in PBS containing mouse anti-c-Fos primary antibody (Santa Cruz Biotechnology, catalog #sc-271243; 1:200). After rinsing in PBS, sections were incubated with biotinylated donkey anti-mouse IgG secondary antibody (Jackson ImmunoResearch Laboratories, catalog #715-065-151; 1:500). The signal was subsequently amplified using the Vectastatin ABC Elite reagents (Vector Laboratories) according to the manufacturer's instructions. Immunolabeling was visualized using a nickel-enhanced diaminobenzidine reaction in the presence of hydrogen peroxide, yielding a blue–black nuclear precipitate in c-Fos^+^ cells. Finally, sections were rinsed, mounted onto gelatin-coated slides, air-dried, and coverslipped.

### Image analyses

To evaluate the neuronal activation levels during the active avoidance retest in the CON and CORT groups, the c-Fos^+^ neurons were sampled bilaterally in the mPFC, NAc, BLA, and hippocampus. Within the dorsal hippocampus, only the dCA3 subregion (dentate gyrus excluded) was analyzed. The images were taken with a digital camera on a microscope (Nikon ECLIPSE Ni-U). Cell counting was manually performed by an observer blinded to group assignment. The sampled PL and IL were selected at the level of AP +3.00 mm from the bregma. The sampling area for the PL was 800 × 800 μm, whereas the sampling area for the IL was 500 × 500 μm. The sampled NAc core (NAcc) and NAc shell (NAcs) were selected at the level of AP +1.80 mm from the bregma, with a sampling area of 500 × 500 μm. The dCA3 and BLA were sampled at the level of AP −2.92 mm from the bregma. The sampling area for the dCA3 was 800 × 800 μm, whereas the sampling area for the BLA was 500 × 500 μm. Examples of c-Fos^+^ neurons in the sampled areas are shown in Figure 2.

**Figure 2.**
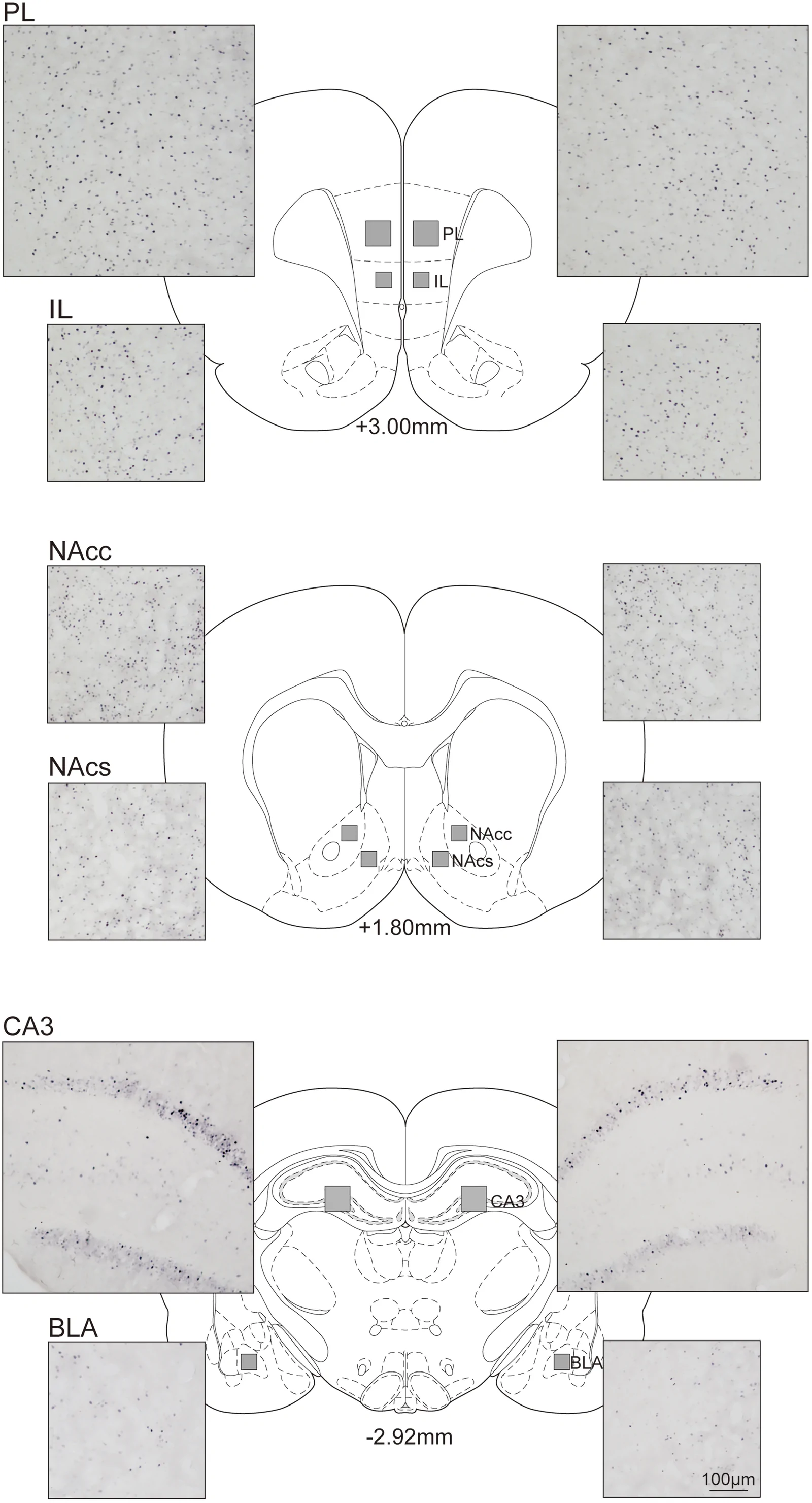
Examples of c-Fos^+^ neurons in the mPFC, NAc, hippocampus, and BLA from a rat in CORT group. Within the hippocampus, only the dCA3 subregion (dentate gyrus excluded) was analyzed. Schematic coronal section at approximately +3.00 mm from the bregma, with images showing sampled c-Fos^+^ neurons in the PL and IL subregions of the mPFC (top panel). Schematic coronal section at approximately +1.80 mm from the bregma, with images showing sampled c-Fos^+^ neurons in the core (NAcc) and shell (NAcs) of the NAc (middle panel). Schematic coronal section at approximately −2.92 mm from the bregma, with images showing sampled c-Fos^+^ labeling in the dCA3 and BLA (bottom panel). Scale bar, 100 μm.

### Statistical analyses

The changes in body weight and fur coat state during the 21 d of chronic corticosterone administration were analyzed using two-way repeated–measure analyses of variance (RM ANOVA) with “Groups” (CON vs CORT) as the between-subject factor and “Days” as the within-subject factor. The percentage of time spent in the open arms in EPM and the immobility time in FST were analyzed with a Student's *t* test. The number of arm entries in EPM was analyzed using a two-way RM ANOVA with “Groups” (CON vs CORT) as the between-subject factor and “Arm Type” (Open vs Closed) as the within-subject factor. The percentage of sucrose intake for SPT was analyzed using a two-way RM ANOVA with “Groups” (CON vs CORT) as the between-subject factor and “Days” as the within-subject factor.

In the active avoidance test, several behavioral parameters were assessed, including the percentage of successful trials, avoidance latency, cued freezing time, the number of feces pellets left in the chambers during the session, and the total number of shocks received during the session. For all the analyses, “Pre” referred to performance after successful training (performance on the third day of the consecutive 3 d of successful avoidance) and “Post” represented performance following chronic corticosterone administration. Each of the parameters was analyzed using two-way RM ANOVA with “Groups” (CON vs CORT) as the between-subject factor and “Sessions” (Pre vs Post) as the within-subject factor.

For c-Fos^+^ cell counts, the cell density (count/mm^2^) in respective sampled regions was analyzed using two-way RM ANOVA with “Groups” (CON vs CORT) as the between-subject factor and “Hemisphere” (left vs right) as the within-subject factor.

For ANOVA analyses, all significant *F*-ratios were reported, followed by Tukey's honestly significant difference (HSD) post hoc tests when significant effects (*p* < 0.05) were detected. The effect sizes were reported as partial eta squared (*η*^2^*_p_*). As an effect size, *η*^2^*_p_* values >0.01, 0.06, and 0.14 were interpreted as indicating small, medium, and large effects, respectively (Richardson, 2011). Data were presented as mean ± SEMs.

## Results

A total of 24 rats were used in this experiment. Three rats failed to reach the performance criterion during training and were excluded from further analyses. For the remaining 21 rats, it took them an average of 15.4 ± 1.0 daily sessions (range from 6 to 26 sessions) to acquire active avoidance performance, with an average successful rate of 93.6 ± 1.4% (“Pre” performance). The 21 rats were assigned to matched control group (CON, *n* = 10) and corticosterone-treated group (CORT, *n* = 11) based on their body weight and their shock intensity used during training before the start of the stress procedure. For the CON group, they had an averaged body weight of 460.3 ± 7.3 g (range from 399 to 525 g), and the averaged shock intensity for training was 0.46 ± 0.03 mA (range from 0.25 to 0.7 mA). For the CORT group, they had an averaged body weight of 470.7 ± 6.9 g (range from 410 to 531 g), and the averaged shock intensity for training was 0.45 ± 0.03 mA (range from 0.25 to 0.7 mA).

We first monitored the physiological changes of the rats during the 21 consecutive days of corticosterone administration. Chronic corticosterone administration significantly affected the gaining of body weight (Fig. 3*A*). There were significant main effects of “Groups” (*F*_(1, 19)_ = 7.20; *p* = 0.02; *η*^2^*_p_* = 0.28) and “Days” (*F*_(20, 380)_ = 2.45; *p* = 0.001; *η*^2^*_p_* = 0.11) and a significant two-way interaction between “Groups” and “Days” (*F*_(20, 380)_ = 56.76; *p* < 0.001; *η*^2^*_p_* = 0.75). Post hoc analyses revealed that the difference in body weight between the two groups reached statistical significance starting from Day 8 (*p*s < 0.05). Chronic corticosterone administration also led to worsening of fur condition (Fig. 3*B*). There were significant main effects of “Groups” (*F*_(1, 19)_ = 68.39; *p* < 0.001; *η*^2^*_p_* = 0.78) and “Days” (*F*_(20, 380)_ = 14.96; *p* < 0.001; *η*^2^*_p_* = 0.44) and a significant two-way interaction between “Groups” and “Days” (*F*_(20, 380)_ = 15.62; *p* < 0.001; *η*^2^*_p_* = 0.45). Post hoc analyses revealed that the difference in fur condition between the two groups reached statistical significance starting from Day 2 (*p*s < 0.05). Together, these results indicated that chronic corticosterone administration was associated with progressive physiological deterioration, reflected by lost in body weight and worsened fur condition over time.

**Figure 3.**
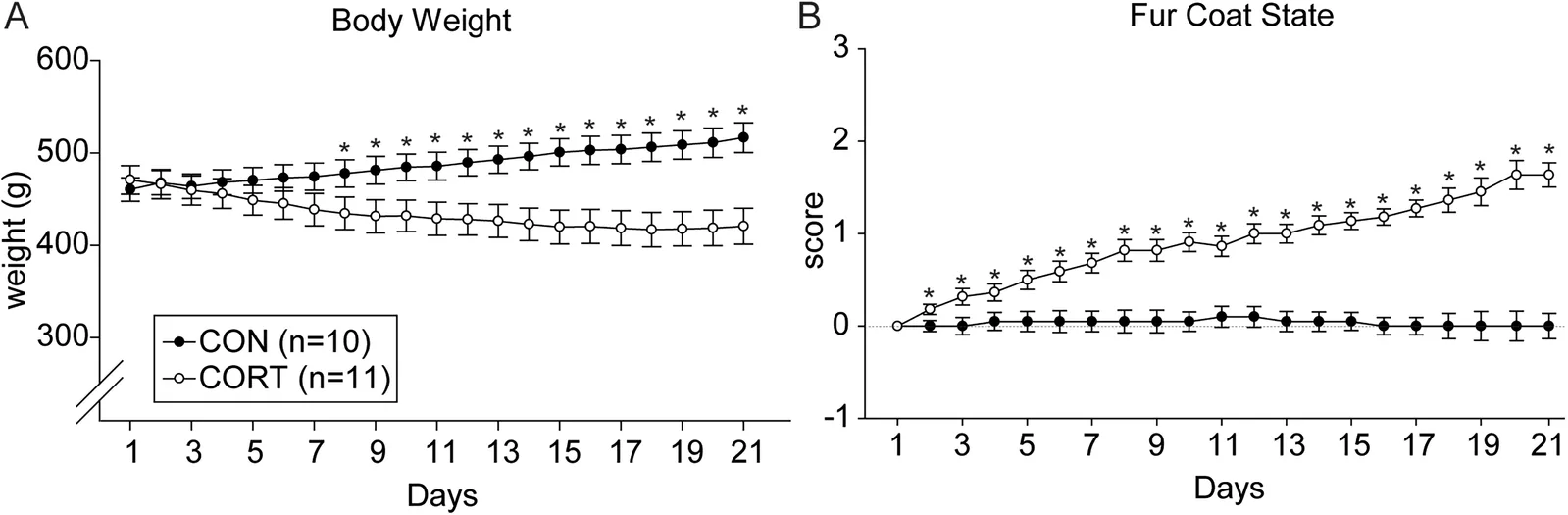
Chronic corticosterone administration had negative impacts on body weight gain and fur coat condition in rats. ***A***, Changes in body weight in the CON and CORT groups over 21 d of corticosterone administration. The CORT group showed a slight and slow decline in body weight over time, with significant differences between the two groups emerging from Day 8. ***B***, Scores of fur coat state for CON and CORT groups over the same 21 d period. The CORT group showed deteriorating fur condition over time, with significant differences between the two groups starting from Day 2. CON, control; CORT, chronic corticosterone administration. (*p < 0.05).

We next evaluated the emotional states of the rats after chronic corticosterone administration. For EPM, there were no statistical differences between the CON group and the CORT group in terms of their percentage of time spent in the open arms (*t*_(19)_ = 0.53; *p* = 0.61). As for the number of entries into the open versus closed arms, there were significant main effects of “Groups” (*F*_(1,19)_ = 6.08; *p* = 0.02; *η*^2^*_p_* = 0.24) and “Arm Type” (*F*_(1, 19)_ = 150.19; *p* < 0.001; *η*^2^*_p_* = 0.89) and a significant two-way interaction between “Groups” and “Arm Type” (*F*_(1, 19)_ = 5.58; *p* = 0.03; *η*^2^*_p_* = 0.23; Fig. 4*A*). Post hoc analyses revealed that the CORT group made significantly fewer entries into the closed arms than the CON group (*p* < 0.05), whereas the number of open-arm entries were low and did not differ between groups. For FST, the CORT group spent more time in immobility (*t*_(19)_ = 11.19; *p* < 0.001; Fig. 4*B*). For SPT, there were significant main effects of “Groups” (*F*_(1,19)_ = 30.44; *p* < 0.001; *η*^2^*_p_* = 0.62) and “Days” (*F*_(2, 38)_ = 8.75; *p* = 0.001; *η*^2^*_p_* = 0.32) and a significant two-way interaction between “Groups” and “Days” (*F*_(2, 38)_ = 6.78; *p* = 0.003; *η*^2^*_p_* = 0.26), suggesting that the CORT group consumed less sucrose solution in general (Fig. 4*C*). Post hoc analyses revealed that the amount of sucrose solution consumed further declined following the first day of test and reached a significant level (both *p*s < 0.05) for the CORT group, but there was no such decline for the CON group. Together, these results suggested that rats underwent chronic corticosterone administration showed hypolocomotion in EPM with fewer entries into the closed arm; however, there was no evidence indicating the differences in anxiety level between the groups as the percentage of time spent in open arms and the number of entries into the open arms were low in both groups. Moreover, chronic corticosterone administration led to an increase of behavioral despair in FST and induced anhedonia of the animals.

**Figure 4.**
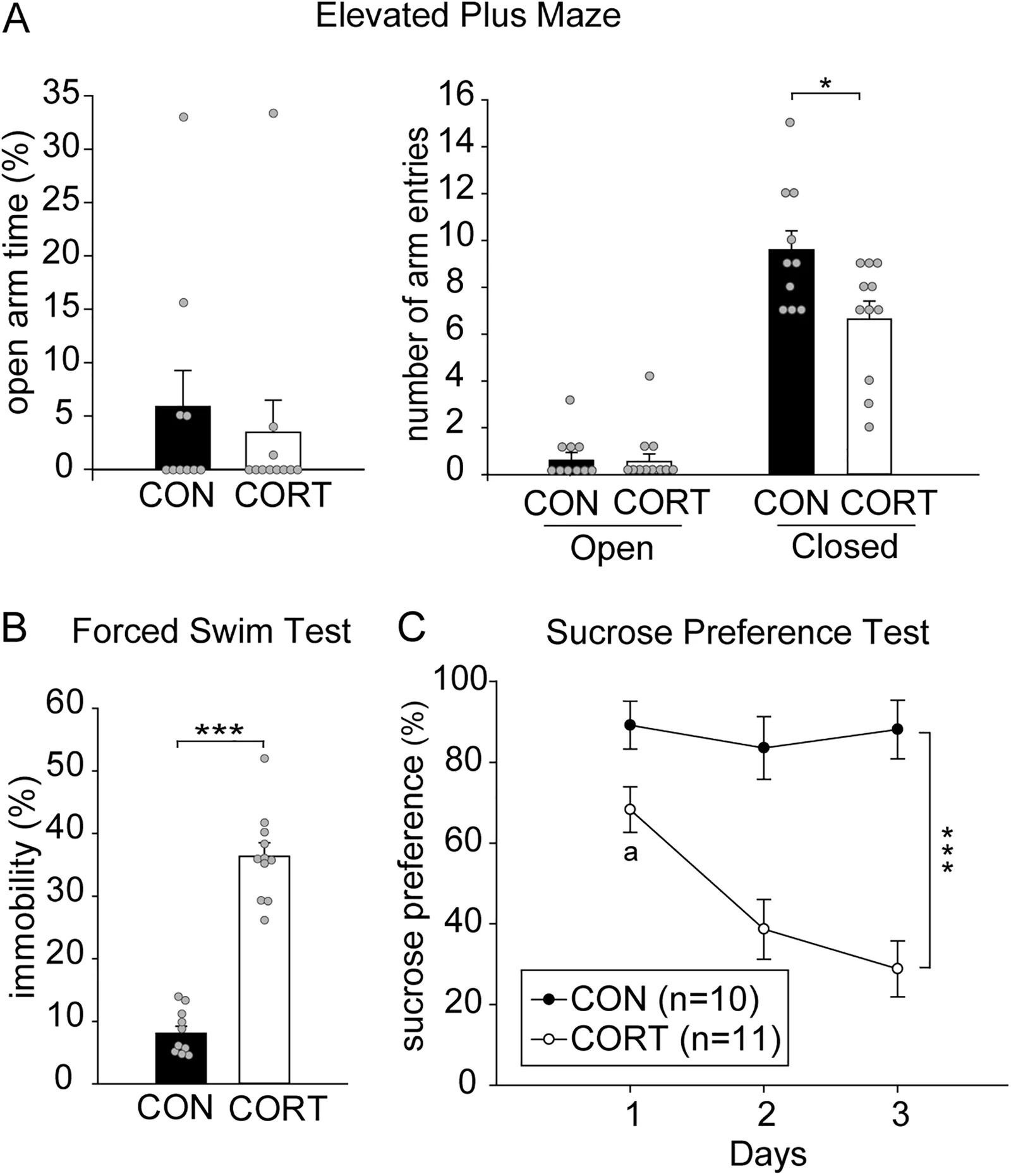
Effects of chronic corticosterone administration on anxiety-like behavior, despair-like behavior, and anhedonia. ***A***, In the EPM task, there was no statistical difference between the CON and CORT groups in the percentage of time spent in the open arms (left panel). There was no statistical difference in the number of open-arm entries between the groups, but the CORT group made significantly fewer closed-arm entries than the CON group, suggesting an overall decrease in locomotion (right panel). ***B***, In FST, the CORT group displayed significantly higher immobility than the CON group, indicating increased despair-like behavior. ***C***, In SPT, the CORT group consumed significantly less sucrose solution compared with the CON group and further declined in time, reflecting the development of anhedonia. CON, control; CORT, chronic corticosterone administration (a, comparisons between Day 1 vs Day 2 or Day 3 in CORT group; ***p < 0.001).

We lastly examined whether the coping strategies of the rats to signaled threats were affected after chronic corticosterone administration. There were no statistical differences in the main effects of “Groups” or the two-way interaction between “Groups” and “Sessions” (all *p*s > 0.05) in any of the behavioral parameters we measured (Fig. 5). However, their overall behavioral performance in active avoidance improved with the passage of time. There were significant main effects of “Sessions” in the parameter of “latency to avoidance” (*F*_(1, 19)_ = 25.92; *p* < 0.001; *η*^2^*_p_* = 0.58) and “cued freezing time” (*F*_(1, 19)_ = 20.66; *p* < 0.001; *η*^2^*_p_* = 0.52). There were marginal main effects of “Sessions” in the parameter of “successful rate” (*F*_(1, 19)_ = 4.19; *p* = 0.06; *η*^2^*_p_* = 0.18) and “number of shocks received” (*F*_(1, 19)_ = 3.26; *p* = 0.09; *η*^2^*_p_* = 0.15). Together, the results suggested that with the passage of time and regardless of the history of chronic corticosterone administration or not, these rats were faster in responding to cues with shorter latency to action, higher successful rates in active avoidance accompanied with lower freezing time to cues, and therefore the fewer number of footshocks they received.

**Figure 5.**
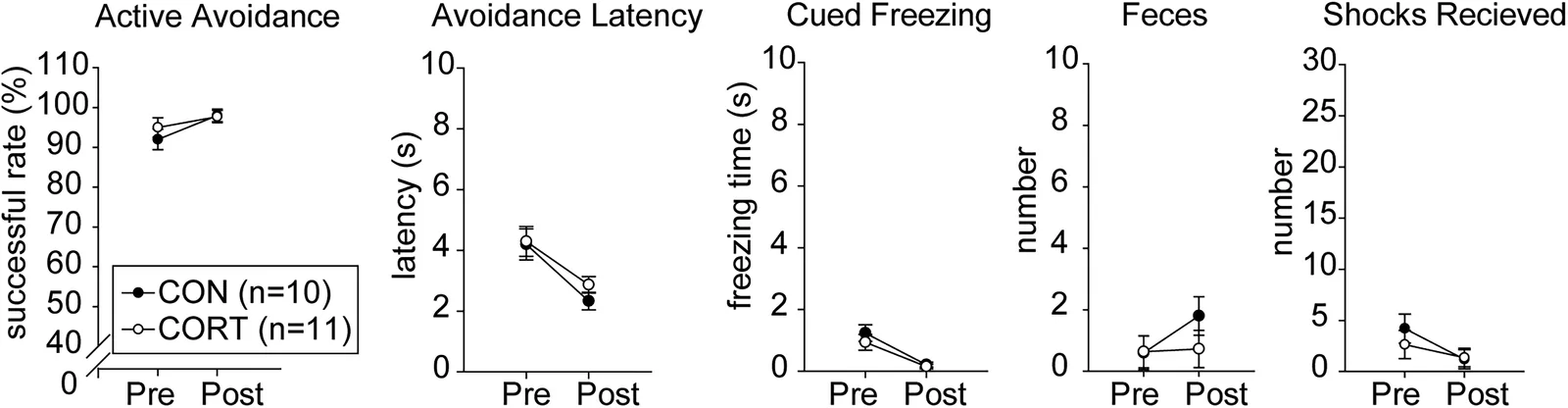
Chronic corticosterone administration did not alter active avoidance performance in successful avoidance rate, avoidance latency, cued freezing time, number of fecal pellets left in the chambers, and number of shocks received during active avoidance tests. “Pre” indicates performance after successful training and “Post” indicates performance following 21 d of corticosterone administration (CORT) or handling (CON).

Neuronal activation levels during the active avoidance retest in the CON and CORT groups were evaluated. There were no statistical differences in the main effects of “Groups” or “Hemisphere,” or the two-way interaction between “Groups” and “Hemisphere” (all *p*s > 0.05), in the PL, IL, dCA3, or BLA (Fig. 6). There were marginal main effects of “Hemisphere” in the NAcc (*F*_(1, 19)_ = 3.02; *p* = 0.1; *η*^2^*_p_* = 0.14) and NAcs (*F*_(1, 19)_ = 3.44; *p* = 0.08; *η*^2^*_p_* = 0.15) but no statistical differences in respective “Groups” or their two-way interactions with “Hemisphere” (all *p*s > 0.05). Together, these results indicated that prolonged corticosterone exposure did not alter c-Fos^+^ expression in the examined brain regions, and no differences in neuronal activation levels were observed between the left and right hemispheres.

**Figure 6.**
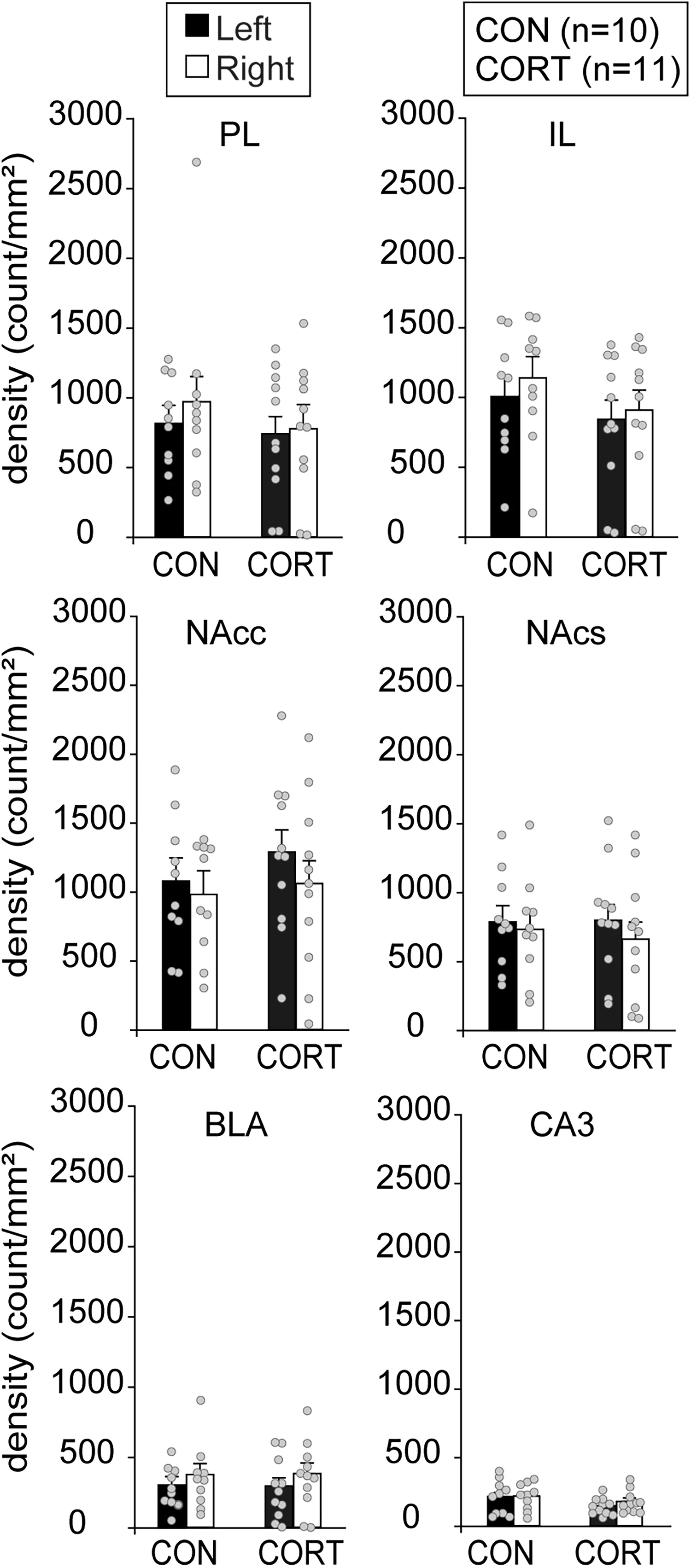
Chronic corticosterone administration did not alter the neuronal activation levels between groups in the prelimbic cortex (PL), infralimbic cortex (IL), nucleus accumbens core (NAcc), nucleus accumbens shell (NAcs), basolateral amygdala (BLA), or dorsal CA3. Left and Right, the two hemispheres. CON, control; CORT, chronic corticosterone administration.

## Discussion

In this experiment, we examined how chronic corticosterone administration to rats affected their physical and emotional states, active avoidance behavior, and the neuronal activation levels in the mPFC, NAc, BLA, and dCA3. Our findings complemented the evidence that chronic corticosterone exposure led to physiological and behavioral changes similar to those seen in human stress-related disorders (Pizzagalli, 2014; Bauer et al., 2022). For the CORT rats, we observed a gradual decline in body weight over time, deterioration in fur condition, and the development of hypolocomotion, despair-like behavior, and anhedonia. However, chronic corticosterone administration did not change the coping strategies of the rats to signaled threats as their performance in active avoidance behavior was not affected. In line with the behavioral results, neuronal activation levels during active avoidance retest showed no differences in CORT rats compared with CON rats in the brain regions examined.

Our corticosterone-treated rats exhibited a slight and gradual decrease in body weight along with a deterioration in fur coat condition over time compared with the control animals. These findings were consistent with previous studies that chronic corticosterone administration induced a range of behavioral and physiological alterations in rodents, including disrupted metabolism and reduced grooming behavior (Nollet et al., 2013; Shin et al., 2024). In EPM, we did not observe differences between the CORT and CON groups in their percentage of time spent in the open arms or the number of entries into the open arms. This finding is consistent with a previous study reporting similar outcomes (Liu et al., 2016) but different from others showing reduced the number of entries into the open arms and increased anxiety-like behaviors following chronic stress exposure (Hata et al., 2001; Bondi et al., 2008). The discrepancy might be attributed to the different parameters used in the chronic stress procedures, for example, the frequency and intensity of the stressors (Buynitsky and Mostofsky, 2009). However, the majority of the animals from both groups in our study exhibited very low numbers of open-arm entries, suggesting a potential floor effect in the EPM to examine the difference in anxiety level due to corticosterone treatment. On the other hand, we observed a reduced number of closed-arm entries in the CORT group, indicating the development of hypolocomotion. In FST, the CORT group exhibited a significant increase in immobility time, supporting the idea that prolonged exposure to stress enhances despair-like behavior, and is in line with findings from previous studies (David et al., 2009; Dieterich et al., 2019). In addition, the SPT revealed a significant reduction in sucrose consumption in the CORT group, which is also consistent with earlier studies demonstrating the effectiveness of chronic stress in inducing anhedonia in rodents (Mao et al., 2022).

Although several of the physiological and behavioral parameters were negatively impacted by chronic corticosterone administration as summarized above, our results did not reveal differences between the CORT and CON groups in their coping strategies to signaled threats in all the parameters we measured, including the successful rate of active avoidance, avoidance latency, cued freezing time, the number of feces left in the chambers after test, or the number of footshocks they received. It is worth noticing that the rats were well trained before the start of chronic corticosterone administration in this study. Repeated training may have promoted a shift from goal-directed avoidance to more habitual stimulus–response behavior. Indeed, previous studies have demonstrated that overtraining reduces behavioral sensitivity to outcome value and stress modulation, reflecting the transition toward habit-based control (de Wit and Dickinson, 2009; Schwabe and Wolf, 2011). Habitual responses are generally more resistant to emotional and stress-related perturbations compared with flexible, goal-directed actions (Schwabe and Wolf, 2009; Perez, 2022). Therefore, chronic corticosterone exposure may have been insufficient to disrupt a well-consolidated avoidance response. The result is consistent with a previous report that chronic restraint stress did not affect active avoidance behavior in rats (Kuan and Chang, 2024). However, the results did not support the idea that individuals may favor passive coping strategies under chronic stress condition (León et al., 2009). Aligned with the behavioral results, we also did not detect differential neuronal activation levels in the brain areas we examined due to chronic corticosterone administration. Among the regions examined, previous studies have suggested the effects of chronic corticosterone administration on the hippocampus; the ventral hippocampus (vHPC) plays a prominent role in emotional regulation and active avoidance behavior (Oleksiak et al., 2024), and chronic corticosterone exposure may exert greater effects on gene expression and neural activity in the vHPC than in the dorsal hippocampus (Caradonna et al., 2022). Limited due to the tissues being processed, we did not detect differences in c-Fos expression in the dCA3 between the CORT and CON groups. Nonetheless, the absence of changes in dCA3 activation level does not rule out the potential involvement of other hippocampal subregions.

Previous studies have shown that sustained corticosterone exposure suppresses ACTH secretion, disrupts the normal circadian rhythm of corticosterone release, reduces adrenal gland weight, and markedly attenuates HPA responses to subsequent stressors (Young et al., 1995; Meijer et al., 2023). The prolonged corticosterone administration in our study may have suppressed the endogenous HPA axis activity through negative feedback mechanisms. Therefore, the behavioral and neuronal effects observed in the present study may reflect not only the consequences of elevated glucocorticoid exposure but also the compensatory adaptations within the HPA axis. Sex differences in stress responsivity have also been reported in humans and rodents, with males and females exhibiting distinct HPA axis responses and differential vulnerability to stress-related psychiatric disorders (Heck and Handa, 2019; Hodes et al., 2024). In rodent studies, for example, female Wistar rats displayed a reduction in two-way active avoidance behavior following repeated stress (Gamaro et al., 1999). Additionally, male Sprague Dawley (SD) rats exhibited reduced active burying behavior and increased immobility after being subjected to chronic plus acute prolonged stress (Roth et al., 2012). Together, these findings suggest that stress-related behavioral adaptations may differ between sexes. Beyond the sex differences, several rat strains have been used in stress research, with SD and Wistar rats being the most common. In our study, we used LE rats, which may partly explain the behavioral outcomes observed. Previous studies have reported strain-dependent differences in stress reactivity and HPA axis regulation. For example, chronically stressed LE rats exhibit lower corticosterone responses to acute stressors compared with SD rats, and LE rats generally display lower baseline anxiety levels (Bielajew et al., 2002; Bagley et al., 2019). Additionally, LE rats have been reported to show habituation following repeated stress exposure (Gregus et al., 2005). Strain-related differences may contribute to the relative preservation of active avoidance performance observed in our study.

We acknowledged a shortcoming in our experimental design that a separate vehicle group was not included; our control animals were handled daily, and all procedures were matched except for corticosterone administration. This approach was used in order to avoid injections per se served as a potential stressor. However, because corticosterone was dissolved in sesame oil containing 10% ethanol, the potential contribution of repeated vehicle exposure cannot be completely ignored. Previous studies have shown that ethanol exposure can influence HPA axis activity, alter corticosterone responses, and modulate fear and avoidance-related behavior (Seeley et al., 1996; Quiñones-Laracuente et al., 2015; Lguensat et al., 2021). Nonetheless, previous report demonstrated that chronic corticosterone paradigms like the one used here have consistently demonstrated that vehicle treatment alone did not induce depressive-like or avoidance-related behavioral alterations (Johnson et al., 2006). Therefore, although vehicle-related effects cannot be excluded, the observed behavioral changes are more likely attributable to sustained glucocorticoid elevation.

To conclude, chronic corticosterone administration induced physiological and affective alterations, including reduced body weight gain, deterioration of fur condition, hypolocomotion, increased behavioral despair, and anhedonia. However, no differences between groups were detected in active avoidance performance or c-Fos expression in the brain regions examined under the present experimental conditions. Given the potential influences of overtraining, HPA axis adaptation, sex- and strain-related variability, and the absence of a vehicle-treated control group, future studies should further investigate the effects of chronic corticosterone exposure on the acquisition and expression of active avoidance behavior in both male and female animals. Integrating these variables into the experimental designs will be crucial for a comprehensive understanding of the neurobiological underpinnings of stress-related conditions.

## Synthesis

Reviewing Editor: Michael McDannald, Boston College Decisions are customarily a result of the Reviewing Editor and the peer reviewers coming together and discussing their recommendations until a consensus is reached. When revisions are invited, a fact-based synthesis statement explaining their decision and outlining what is needed to prepare a revision will be listed below. The following reviewer(s) agreed to reveal their identity: Gessynger Morais-Silva.

Dear Authors,

Thank you for submitting your manuscript "Chronic Corticosterone Administration Induced Affective Disturbances, but Did Not Alter Consolidated Active Avoidance Coping or Neuronal Activation Level, in Male Long-Evans Rats" to eNeuro. Both reviewers found the topic relevant and the methodology clearly described. However, following a consultation between the reviewers and the handling editor, a number of concerns have been identified that require attention. These are summarized below.

1. Terminology. The most pervasive issue in the manuscript is the conflation of chronic corticosterone administration with chronic stress throughout, including in the abstract, results, and discussion sections. These are not equivalent constructs and cannot be used interchangeably. The manuscript must be systematically revised to accurately reflect that the manipulation was pharmacological corticosterone administration. Where comparisons to the chronic stress literature are made, these should be explicitly framed as contextual rather than direct equivalents. The introduction should also be expanded to address the distinction between chronic stress, glucocorticoid elevation, and mood-related disorders more carefully.

2. EPM data and interpretation. Figure 3A reveals that a large proportion of animals in both groups had zero or near-zero open arm entries. Closed-arm entries must be reported as an index of locomotor activity, as their absence makes it impossible to determine whether the null anxiety result reflects a true absence of anxiety-like behavior or general hypoactivity. The authors should also engage more directly with the floor-level EPM performance in their interpretation, rather than relying solely on the LE strain explanation in the discussion.

3. Active avoidance conclusion and data reporting. By the Post timepoint, both groups are performing near ceiling across all avoidance parameters (Figure 4). The conclusion that chronic corticosterone does not alter coping strategies is overstated in this context. The authors are asked to report more granular within-session data, such as trial-by-trial or time series performance across the session, if available. This would allow a more sensitive assessment of whether group differences exist at a finer timescale than session-level averages currently capture. Additionally, the overtraining and habit formation argument in the discussion should be developed as a genuine limitation of the study rather than a defense of the null result, and the framing of this conclusion in the abstract and discussion must be softened accordingly.

4. Vehicle and ethanol confound. The control group did not receive injections, and the corticosterone solution contained sesame oil with 10% ethanol. The potential contribution of chronic ethanol exposure to the behavioral results is not discussed. The authors should acknowledge this as a limitation and address it in the context of existing literature on vehicle controls in chronic corticosterone paradigms.

5. Hippocampal c-Fos. If brain tissue sections containing the hippocampus were retained from the original animals, the authors are asked to include hippocampal c-Fos analysis given the well-established role of this region in chronic corticosterone effects. If tissue is not available, the absence of hippocampal data should be explicitly acknowledged as a limitation in the discussion.

6. HPA axis feedback. The authors should discuss whether 21 days of exogenous corticosterone at the dose used is likely to have suppressed endogenous HPA axis activity via negative feedback, and what implications this may have for interpreting the behavioral and neural findings.

7. Image analysis reporting. The methods section should be expanded to specify whether experimenters were blinded to group assignment during c-Fos counting, and to report the number of sections analyzed per animal.

8. Sex as a biological variable. The justification for using male rats only should be stated more explicitly, and the limitation this places on the generalizability of the findings should be addressed with greater depth in the discussion.

9. Minor points. The behavioral apparatus subsection should be retitled to reflect the full range of equipment used in the study. The conclusion section should be revised to reflect the actual findings rather than primarily orienting toward future directions.

The authors are asked to provide a detailed point-by-point response addressing each of the concerns above alongside any revised manuscript.
